# COVID-19 or threat of a nuclear war in Europe? A cross-sectional study of anxiety levels in adults living in Portugal

**DOI:** 10.3389/fpubh.2023.1159172

**Published:** 2023-07-31

**Authors:** Filipe Prazeres, Tiago Maricoto, Inês Sampaio Lima, Pedro Simões, José Augusto Simões

**Affiliations:** ^1^Faculty of Health Sciences, University of Beira Interior, Covilhã, Portugal; ^2^Family Health Unit Beira Ria, Gafanha da Nazaré, Portugal; ^3^CINTESIS@RISE, MEDCIDS, Faculty of Medicine of the University of Porto, Porto, Portugal; ^4^Personalized Health Care Unit Fundão, Fundão, Portugal

**Keywords:** COVID-19, pandemic, mental health, anxiety, nuclear war, Portugal

## Abstract

**Background:**

Since 2019, Europe has experienced ongoing stressors with the emergence of the COVID-19 pandemic and the Russian–Ukrainian War, which have had social, financial, physical, and psychological impacts. Studies suggest that anxiety, fear, post-traumatic stress disorder, depression, and other psychological disorders are common in such situations, and there is a need for more research on the impact of the war on mental health in Portugal. The main goal of the present study was to assess the impact of the fear of COVID-19 and anxiety related to nuclear war on the general anxiety levels of adult individuals living in Portugal.

**Methods:**

A cross-sectional study was conducted from May to July 2022 using an online questionnaire built on the Google Forms platform. Portuguese-speaking male and female individuals aged 18 years or older, who provided informed consent and agreed to participate, were included. The outcome variable was defined using the Portuguese version of the GAD-7 scale, while the main predictors were the FCV-19S and the NWA Scale in Portuguese. Linear and logistic regression models were used to test associations between predictors and outcome variable.

**Results:**

The study included 1,182 participants, with a mean age of 46.5 (±11.7) years, mostly women (80.6%). The global mean GAD-7 score was 5.8 (±4.5) points, and 17.9% of the participants scored above the 10-point cutoff. Higher scores were found in both the FCV-19S and the NWA scale among participants with anxiety, as measured by both a 10-point cutoff (*p* < 0.001), and GAD-7 scale mean scores (*p* < 0.001). The study showed that fear of COVID-19 [OR of 1.133 (95%CI: 1.097–1.170)] and, at a lesser extent, nuclear war anxiety [OR of 1.020 (95%CI, 1.009–1.031)] contribute to anxiety in the general population. This is also true for those with a personal history of anxiety, revealed by multiple regression.

**Discussion:**

This study contributes to the research on COVID-19’s impact on anxiety and provides the first comprehensive assessment of nuclear war anxiety in Portugal. Results highlight the need for long-term care for anxiety, as prevalence is expected to increase due to the pandemic and war, even in non-conflict areas like Portugal.

## 1. Introduction

Pandemics, as well as wars, are well-documented stressors with social, financial, physical, and particularly psychological impacts (1–4). Since 2019, the world, particularly Europe, has experienced continuous stress factors with the emerging of SARS-COV2 pandemic and, shortly after, the Russian–Ukrainian War.

The SARS-COV2 pandemic, first identified in Wuhan, China, in December 2019 and quickly expanded to a worldwide scale (5). It was declared a pandemic by the World Health Organization on 11th March 2020 (6), and the first case in Portugal was reported on 2nd March 2020 (5). Governments worldwide, especially in Europe, instituted measures to minimize the transmission and impact on health and the economy during the pandemic. The Portuguese Government’s response was swift, including various restrictions such as sanitary fences, closure of commercial establishments and educational institutions, and mandatory confinement at home for various periods (5). At the end of December 2020, the first vaccine against COVID-19 was administered at Centro Hospitalar Universitário de São João, in Porto, Portugal, marking the beginning of the vaccination plan (5), and the gradual decrease in mortality (7). However, the situation in healthcare was described as unsustainable in January 2021 (5), with the main peak between October 2020 and March 2021 (8), followed by another peak in mortality between December 2021 and March 2022 (8). By the end of April 2022, it is estimated that there were more than 65 thousand daily infections and more than 22 thousand deaths due to COVID-19 in Portugal (9).

On the other hand, the Russian–Ukrainian War, beginning on 24 February 2022, rapidly emerged as the biggest humanitarian emergency in Europe since the Second World War (2). Although Ukrainians are the most affected, with millions of refugees, death of family or friends, and damage to patrimony (2), the economic and psychological impact of the war in Europe cannot be disregarded. Among other threats, the fall of financial markets around the world and the rise of prices for oil, natural gas, metals and food products (3, 10), and it introduced the possibility of a nuclear war (11).

Some studies suggest that anxiety, defined as fear or nervousness about what might happen, may be exacerbated in stressful or threatening situations such as wars and pandemics. Anxiety and fear, as well as post-traumatic disorder, depression and other psychological disorders are well documented in previous pandemics (12–15) and wars (1, 16), especially among soldiers and survivors (1).

The outbreak of COVID-19 has led to stressful situations worldwide, and there are several studies on the mental health concerns related to the virus (17–22). In Portugal, the literature also highlights the impact of the COVID-19 pandemic on psychological distress (23–26).

While Ukrainians have been the most impacted by the Russian–Ukrainian War, residents of other countries also experience anxiety and fear (2, 27, 28). A study revealed that Czech university students were highly concerned about the news of the war, with more than one-third of the participants exhibiting moderate and severe anxiety and over two-fifths exhibiting moderate, moderately severe and severe depression (2). Another study reported about 42.1% of the participants felt depressed due to the possibility of nuclear war (28).

A recent population survey of 621 adults in Portugal observed that 77% of the participants were afraid of a nuclear war in Europe following Russia’s invasion of Ukraine, and less than 20% believed that there is no risk for European citizens’ security (29). However, this topic has been less researched compared to the impact of COVID-19 pandemic on societies, and there are no published studies on the impact of the Russian–Ukrainian War on mental health in Portugal.

The main goal of the present study was to assess the impact of the fear of COVID-19 and anxiety related to nuclear war on the general anxiety levels of adult individuals living in Portugal. We hypothesize that demographic and psychological factors, as well as individual experiences and perceptions related to current events like the COVID-19 pandemic and the threat of nuclear war, are associated with anxiety symptoms.

## 2. Materials and methods

### 2.1. Study design, setting, and sample

A cross-sectional study was performed. From May to July 2022, an online questionnaire built into the Google Forms platform was conducted among adults living in Portugal.

In this study were included Portuguese speaking male or female individuals aged ≥18 years, able to read and write in Portuguese, with access to the Internet/email, after giving their informed consent and agreed to participate. Individuals were recruited by receiving the web link of the questionnaire through the research team’s network of contacts, through community groups in social networks, and through other participants by snowball sampling.

Taking into account that in Portugal reside 10,343,066 individuals (30), the minimum required sample size of *n* = 385 was calculated for proportions, considering the most conservative scenario (a proportion of 50%), a level of confidence of 95% and an error margin of 5%.

The present study observed the Declaration of Helsinki ethical principles for medical research involving human subjects and was approved by the Ethics Committee of the University of Beira Interior – Portugal (CE-UBI-Pj-2022-038-ID1367). Electronic consent was obtained from all subjects involved in the study. Responses to the electronic questionnaire were anonymous.

The STROBE guidelines were used to ensure the reporting of this cross-sectional study (31).

### 2.2. Data collected by the questionnaire and study variables

The main predictors were defined as the Fear of COVID-19 Scale and Nuclear War Anxiety Scale in Portuguese.

Fear of COVID-19 Scale (FCV-19S): seven items rated on a five-point Likert scale (ranging from 1 = strongly disagree to 5 = strongly agreeing), such as “I am most afraid of coronavirus-19,” “It makes me uncomfortable to think about coronavirus-19,” “My hands become clammy when I think about coronavirus-19,” “I am afraid of losing my life because of coronavirus-19”; developed by Ahorsu and colleagues in 2020 (32). A recently published systematic review of studies from 21 countries (16 languages, including Portuguese) synthesized the psychometric evidence for the Fear of COVID-19 Scale and found that it is a valid and robust instrument for assessing fear of COVID-19 (33). A total score is calculated by adding up the scores for the individual items, with a range of possible scores from 7 to 35. Higher scores indicate a higher fear of COVID-19. In the present study, the version validated for the Portuguese population by Magano and colleagues in 2021 (34) was used and found to have good internal consistency (Cronbach’s alpha of 0.89).

Nuclear War Anxiety (NWA) Scale: twenty-one questions rated on a seven-point Likert scale (from 1 = strongly disagree to 7 = strongly agree), such as “Eliminating the possibility of a nuclear war should be everyone’s highest priority,” “The threat of a nuclear war haunts my thoughts,” “Eliminating the possibility of a nuclear war is worth any price,” “Considering the prospect of nuclear war, I can find little hope for the future”; developed by Chandler in 1991 (35). A total score is calculated by adding up each item score (ranging from 21 to 147), and the higher the score, the greater the fear of nuclear war.

This scale measuring fear from nuclear war that has been published is the only one of its kind. Since there was no existing Portuguese version of the scale, our team used the translation methodology that we typically follow (36). This methodology, which is based on Brislin’s four standard techniques (37), was used to ensure equivalence between the original and translated measure. The first step was to have a panel of six general practitioners who were native Portuguese speakers and fluent in English translate the English-language items. The translations were then reviewed by the authors who were not part of the initial translation process to ensure accuracy, semantics, and cultural appropriateness. Each item from the scale was evaluated one by one, and the translated item that best retained the original meaning and conveyed it in the simplest form was chosen. Accuracy, semantics, and cultural appropriateness were prioritized during the selection process, and items were adapted where needed. Based on all the inputs, an initial translated version was created for the scale. The final version of the Nuclear War Anxiety (NWA) Scale was back-translated by a native English speaker and matched with the original version. Suitable modifications were made to ensure that the same meaning was conveyed while also retaining cultural appropriateness. The final Portuguese version was then pilot-tested in 20 individuals from the general population who were not part of the survey to ensure language and cultural appropriateness. No alterations to the items were necessary in terms of ambiguity or misinterpretation. In the present study, Cronbach’s alpha (internal consistency) for Nuclear War Anxiety (NWA) Scale was 0.84.

The following covariates were also collected: gender, age, marriage status, educational level, employment status, previous COVID-19 infection, previous anxiety disorder and previous exposure to war zones.

The Generalized Anxiety Disorder (GAD-7) scale in the Portuguese version (38) was used to define the outcome variable. The scale was used as a continuous variable according to crude values, which ranged from 0 up to 21. It was also used as a dichotomous variable (present/absent) using a 10-point cutoff, as sensitivity and specificity exceeded 0.80 (39). This approach allowed us to check the consistency of the detected predictors in both model building strategies.

The Generalized Anxiety Disorder (GAD-7) Scale is a seven item anxiety scale rated on a 4-point Likert scale (from 0 = not at all to 3 = nearly every day) (39), such as “Feeling nervous, anxious, or on edge,” “Not being able to stop or control worrying,” “Worrying too much about different things.” It has already been adapted and validated for the Portuguese population (38). Adding the scores from the items of the scale allows the calculation of a total score. In the present study, Cronbach’s alpha for the Generalized Anxiety Disorder (GAD-7) Scale was 0.90, indicating a high level of internal consistency.

### 2.3. Statistical analysis and model building

All data were analyzed using IBM SPSS Statistics^©^ and STATA Statistical Package^©^ software and the alpha level was set at 0.05. Recommendations from the TRIPOD statement were followed to report multivariable prediction model results (40).

To test associations between predictors and outcome variables, we used linear and logistic regression models. For model building, we first performed bivariate associations in order to identify significant variables to be included at a 0.25 alpha level (41). To check the applicability of parametric tests, normality of continuous variables was assessed using Kolmogorov–Smirnov test. Multivariable models were created using step-up and step-down approaches, and different models were validated using homoscedasticity tests and validation tools.

## 3. Results

1,204 participants were enrolled in the study, but 22 (1.8%) did not answer some questions. Due to this low rate of missing data, we decided to exclude them. Therefore, we included 1,182 participants with complete data, with a mean age of 46.5 (±11.7) years, mostly women (80.6%). Almost all participants were employed (91.7%) and most were married (63.1%). 60.5% had a previous COVID-19 infection, 43.2% had a previous history of anxiety, and only 5.6% had been exposed to a war zone.

Detailed baseline data on demographic and clinical features are reported in Table 1.

**Table 1:** Data from demographic and clinical variables collected from all participants and comparisons according to the major outcome variables.

Predictor variable [Mean ± SD or *N* (%)]	Total	Anxiety*			Generalized Anxiety Disorder (GAD-7) score^*^	
		No	Yes	*p* value	Mean	*p*-value
No. of total participants	1,182	971 (82.1%)	211 (17.9%)	–	5.8 (±4.5)	–
Age	46.5 (±11.7)	47.4 (±11.3)	42.7 (±13.0)	<0.001^†^	–	<0.001^§^
Men	229 (19.4%)	202 (88.2%)	27 (11.8%)	0.008^‡^	4.7 (±4.1)	<0.001^†^
Women	953 (80.6%)	769 (80.7%)	184 (19.3%)		6.0 (±4.5)	
Married	746 (63.1%)	638 (85.5%)	108 (14.5%)	<0.001^‡^	5.4 (±4.3)	<0.001^⁑^
Divorced	145 (12.3%)	117 (80.7%)	28 (19.3%)		6.0 (±5.0)	
Widowed	18 (1.5%)	18 (100%)	0 (0%)		4.0 (±3.2)	
Single	273 (23.1%)	198 (56.7%)	75 (43.3%)		6.8 (±4.7)	
Educ. Elementary school	5 (0.4%)	3 (60%)	2 (40%)	0.007^#^	9.2 (±8.0)	0.003^⁑^
Educ. High school	250 (21.2%)	190 (76%)	60 (24%)		6.5 (±5.0)	
Educ. Superior/College	254 (38.4%)	374 (85.3%)	80 (14.7%)		5.9 (±4.4)	
Educ. Master/Doctorate	273 (40%)	404 (85.4%)	69 (14.6%)		5.2 (±4.2)	
Employed	1,084 (91.7%)	902 (83.2%)	182 (16.8%)	<0.001^#^	5.6 (±4.4)	<0.001^⁑^
Unemployed	22 (1.9%)	16 (72.7%)	6 (27.3%)		7.0 (±6.0)	
Student	46 (3.9%)	25 (54.3%)	21 (45.7%)		9.4 (±5.3)	
Retired	25 (2.1%)	24 (96%)	1 (4%)		4.8 (±3.0)	
Healthcare professional	5 (0.4%)	4 (80%)	1 (20%)		5.2 (±3.1)	
Previous Covid-19 infection	715 (60.5%)	602 (84.2%)	113 (15.8%)	0.024^‡^	5.7 (±4.4)	0.366^†^
No previous Covid-19	467 (39.5%)	369 (79%)	98 (21%)		5.9 (±4.6)	
Previous Anxiety	511 (43.2%)	353 (69.1%)	158 (30.9%)	<0.001^‡^	7.8 (±4.7)	<0.001^†^
No previous Anxiety	671 (56.8%)	618 (92.1%)	53 (7.9%)		4.3 (±3.6)	
Previous War zone exposure	66 (5.6%)	53 (80.3%)	13 (19.7%)	0.74^‡^	5.7 (±4.7)	0.888^†^
No previous War zone exp.	1,116 (94.4%)	918 (82.3%)	198 (17.7%)		5.8 (±4.5)	
Fear of COVID-19 Scale	14.4 (±5.6)	13.5 (±5.2)	18.2 (±6.1)	<0.001^†^	–	<0.001^§^
Nuclear War Anxiety scale	87.5 (±18.9)	85.6 (±18.3)	96.3 (±18.9)	<0.001^†^	–	<0.001^§^

*Outcome data presented as values considering only the count of participants within each of the predictor variables. †Independent samples *t*-test. ‡Chi-squared test. #Fisher exact test. §Pearson correlation test. ⁑Kruskal–Wallis test.

The global mean GAD-7 score was 5.8 (±4.5) points, and 17.9% of the participants showed scores above the 10-point cutoff. This was more pronounced in women versus men (19.3% vs. 11.8% prevalence, *p* = 0.008, chi-squared test = 7.114), single persons (43.3%, *p* < 0.001, chi-squared test = 27.146) and students (45.7%, *p* < 0.001, fisher exact test = 25.219). We also found a trend for lower anxiety prevalence with higher educational levels, from being higher in elementary school levels (40%) to lower in master or doctorate levels (14.6%) (*p* = 0.007, fisher exact test = 11.579). Participants with higher anxiety levels were also younger (mean: 42.7 years (±13.0) vs. 47.4 (±11.3), *p* < 0.001, independent samples *t*-test = −4.859). These trends were also consistent when analyzing GAD-7 mean scores among those subgroups (Table 1).

Higher scores were found, both in Fear of COVID-19 Scale and Nuclear War Anxiety scale, among participants with anxiety, either considering a 10-point cutoff (*p* < 0.001, independent samples *t*-test = 10.311 and 7.616, respectively), or in GAD-7 scale mean scores (*p* < 0.001, Pearson linear correlation = 0.414 and 0.318 respectively) (Table 1).

**Figure 1:**
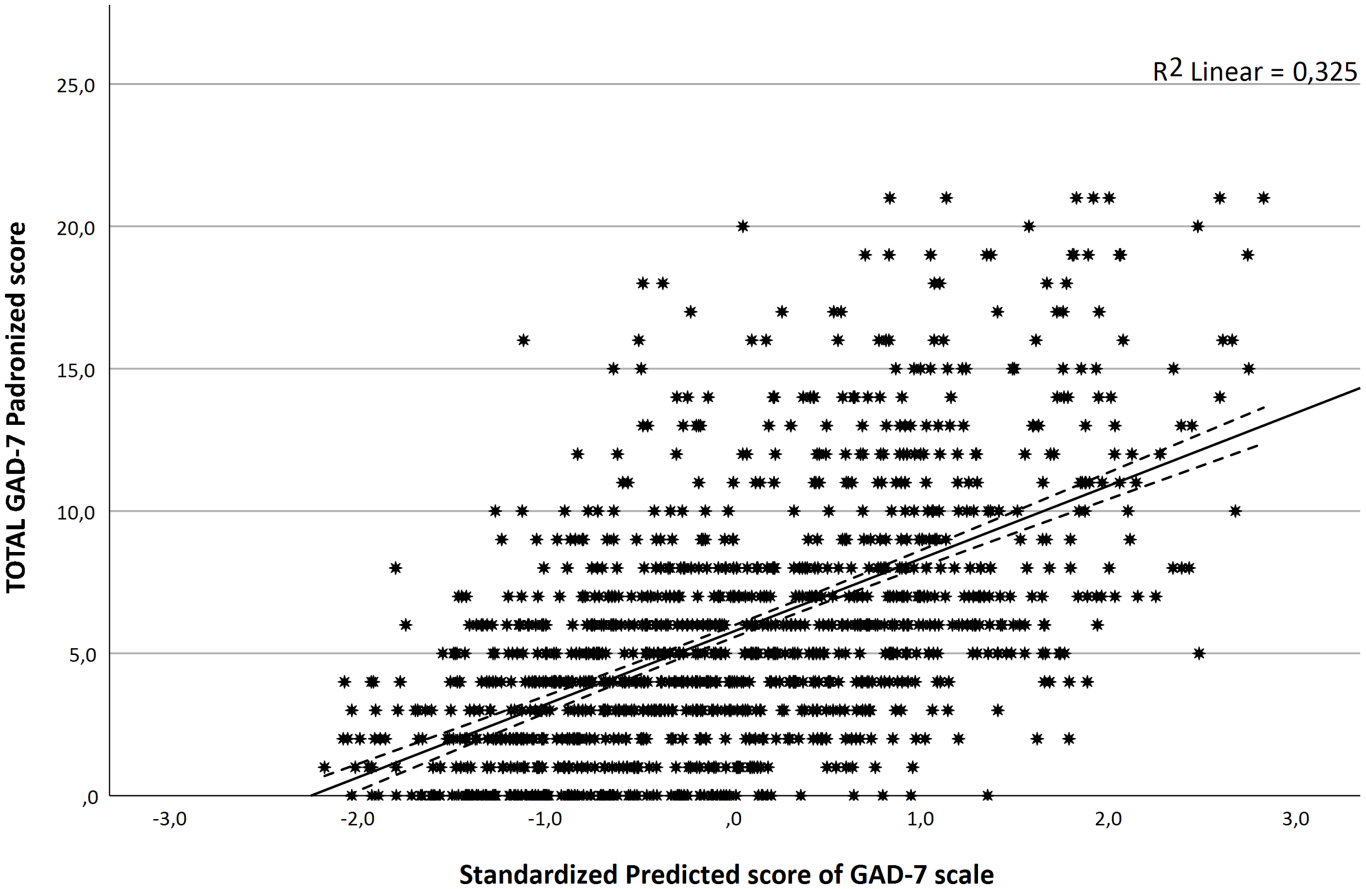
Regression line of the multivariate analysis to GAD-7 score.

Table 2 reports the most relevant results regarding the best-fitted multivariate models with major identified predictors. Figure 1 presents the regression line of the multivariate analysis to GAD-7 score.

**Table 2:** Predictors identified on multivariate analysis according to the major outcome variables.

Outcome – Anxiety (logistic regression)				
Predictor variables in the model	OR	Value of *p*	95%CI	
Age	0.956	<0.001	0.942	0.970
Previous COVID-19 infection	0.597	0.004	0.422	0.844
Previous Anxiety	4.243	<0.001	2.950	6.103
Fear of COVID-19 Scale	1.133	<0.001	1.097	1.170
Nuclear War Anxiety scale	1.020	<0.001	1.009	1.031
(Constant)	0.023	<0.001		
AUC: 0.822 (95%CI: 0.792; 0.852); Nagelkerke Pseudo-*R*^2^: 0.313; Hosmer&Lemeshow *p* = 0.484. This model presented a sensitivity of 27% and a specificity of 96%, a positive predicted value of 59.4% and a negative predicted value of 85.9%.				

Logistic regression for having “Anxiety” identified as major significant predictors: age (OR: 0.956 for each age year), having a previous COVID-19 infection (OR: 0.597), having previous anxiety (OR: 4.243), Fear of COVID-19 Scale (OR: 1.133 per unit increase in questionnaire score), and Nuclear War Anxiety scale (OR: 1.02 per unit increase in questionnaire score).

Linear regression for “GAD-7 score” identified as major significant predictors: age (decrease in 0.064 points for each age year), being employed or a healthcare professional (decrease in 0.971 points), having previous anxiety (increase in 2.591 points), Fear of COVID-19 Scale (increase in 0.246 points for each unit), and Nuclear War Anxiety scale (increase in 0.041 points for each unit).

## 4. Discussion

The present study showed that the prevalence of moderate generalized anxiety symptoms in our sample (17.9%) is similar to the prevalence found in other studies about the impact of COVID-19 pandemic (19–21), but higher than the prevalence of anxiety in Portugal before COVID-19 outbreak (4.9%). When compared to the burden of the Russian–Ukrainian War 2022 (2), our prevalence seems to be lower (17.9% vs. 22.3%). However, this study only analyzed young adults, so the comparison must be made with caution.

As known from the literature, there are sociodemographic characteristics of patients that impact the prevalence of anxiety.

According to gender, we found that anxiety symptoms appear to be more pronounced in women. However, this variable was not present in the logistic or linear regressions. Most of the literature shows a higher prevalence of anxiety in women compared to men (2, 19–22). Some hypotheses in the literature suggest that women’s psychological distress during the COVID-19 pandemic may be due to the negative impact on the workforce, where women represent a large proportion, and due to women’s neurobiological responses to stressors (20, 42, 43). However, a study conducted in China during COVID-19 outbreak showed no differences between genders (44). Studies on gender differences in risk perception have shown that women are more concerned about risks involving their home and family, while men are more concerned about risks involving their working life (e.g., unemployment and economic problems) (45–47). Since the COVID-19 pandemic and Russian–Ukrainian War affect both areas (home/family and working life), it is possible that gender was not present in the logistic and linear regressions because it impacts both genders, but in different ways. Another study also reported a statistical difference in adoption of recommended preventative health behaviours in response to the COVID-19 pandemic according to gender. However, when self-reported fear of the COVID-19 pandemic was included as a predictor, the gender difference stopped being statistically significant (48). Therefore, another hypothesis is that gender could be a confounding factor related to fear of the COVID-19 pandemic.

Patients’ anxiety decreases with the age, which is in line with the literature (19, 20, 22). One previous study (2) did not report statistically significant differences between participants of different age groups, but this study only explored young adults. The impact of COVID-19 pandemic in school closures and social events may have been responsible for students’ emotional distress (20, 49). This is consistent with both our findings and the literature (20, 22), which shows that student status is associated with higher levels of anxiety.

On the other hand, we found that being employed or being a health professional decreases the level of anxiety. We hypothesize that this is due to their lower concern of the impact of the pandemic and/or war on their financial situation. In the case of being a health professional, their greater knowledge of the COVID-19 pandemic may help them process pandemic news in a calmer way.

Additionally, we found that individuals with higher educational levels seem to have a lower prevalence of anxiety. Our findings are in line with the literature (20). The reasons for this are not clear, but we hypothesize that, similar to being a health professional, having more education can help a person better process news/information related to COVID-19 and war, making them less prone to anxiety.

Single people seemed to have a higher prevalence of anxiety. There is no consensus in the literature regarding marital status and the prevalence of anxiety, with some studies (20, 50) finding that being divorced or widowed were predictive factors for symptoms of anxiety, and one study indicating that married status was related with higher levels of anxiety when compared to unmarried individuals (20, 51). It seems that close family members can act as protective or stressful factors for anxiety, which can explain the mixed results in the literature.

Regarding previous COVID-19 infection, we found that having had COVID-19 infection is a protective factor for anxiety. However, this goes against the literature that showed that anxiety was higher in people affected by COVID-19 or who had close contact with COVID-19 infected people (20, 22). One explanation for our findings could be that the majority of people who had COVID-19 infection had mild symptoms or were asymptomatic, so the fear of infection could be reduced since what they experienced was not so bad as reported in the media.

Previous anxious people were more prone to be anxious regarding the fear of COVID-19 infection and nuclear war, which is in line with the literature (20) that mentions that individuals with past or present mental disorders or psychiatric illnesses are more sensitive to external stressors, such as social isolation and media use exposure (20, 52–54).

Anxiety increased as units on the Fear of COVID-19 and Nuclear War Anxiety scales increased. Our findings are in line with the literature, namely regarding the fear of COVID-19, which shows higher anxiety in people that worry about being infected or are more concerned about the war (2, 20).

We could not make inferences about the impact of being exposed to war in anxiety, since only 5.6% of the sample was exposed to it. The literature is unanimous about the impact of exposure to war on the prevalence of anxiety, both in civilians and deployed service members (55, 56).

Regarding the strengths and limitations of the present study, it is valuable to state that this is the first study in Portugal to evaluate the joint impact of two important stressors – the fear of COVID-19 and of a nuclear war – on the general anxiety of the adult population. It is also important to underline that the period when this study was conducted – 6th wave of COVID-19 pandemic and consolidation phase of Russia-Ukraine war – was ideally chosen to better study the role of these dependent variables on anxiety. As limitations, we would like to point out the cross-sectional nature of the study, it is not possible to determine the direction of causality or to make inferences about the effects of one variable on another. Instead, cross-sectional studies are useful for exploring associations between variables, generating hypotheses (57). Therefore, future longitudinal analysis will be needed to further study this phenomenon. Additionally, the low prevalence in the sample of individuals exposed to war conflicts impedes the study of the effect of being exposed to war on anxiety. The sample was recruited using a non-probability sampling method, which may not represent the entire population of interest. Conducting the survey online may have excluded individuals who do not have access to the internet or are not comfortable with completing surveys online. This may limit the generalizability of the study findings. Nonetheless, considering the very low rate of missing data (1.8% of all sample), the risk for non-response bias is low, which strengthens the confidence in the results. Even though a potential bias related to careless responding might exist due to the online nature of the survey (58), we believe this might be attenuated considering the large sample size. Additionally, the total variance extracted by either of the two main factors/predictors of interest does not exceeds 50%, indicating that the probability of a common method bias in our study is very low.

Based on the findings of the present study, several recommendations can be made for various stakeholders. Given that the prevalence of anxiety symptoms was found to be higher among young adults, it is crucial for healthcare professionals to prioritize mental health screening and treatment for this age group. They should also take into account the impact of employment status and previous anxiety history when devising interventions. Healthcare professionals should ensure that accurate information is shared with the public. People should be made aware of the pandemic and war’s impact on their mental health and take steps to manage their anxiety levels, including seeking professional help if needed and avoiding misinformation and sensationalized news. Government and policymakers should ensure that the population has access to mental health resources, focus on providing financial support and resources for those most affected by the pandemic and war, including those who are unemployed. Additionally, the media should provide accurate and reliable information on the current status of the pandemic and the situation in Ukraine to help people better understand the situation and reduce anxiety levels.

## 5. Conclusion

In conclusion, the present study not only adds to the growing body of research about the impact of COVID-19 pandemic in increasing anxiety levels but also provides the first comprehensive assessment of nuclear war anxiety in Portugal, and its association with the general population’s anxiety. This study has shown that both fear of COVID-19 and, at a lesser extent, nuclear war anxiety contribute to the general population’s anxiety. This is also true for those with a personal history of anxiety, as revealed by the multiple regression analysis. These findings suggest that in the current setting of COVID-19 pandemic and Russia-Ukraine war, a key policy priority has to be the long-term care of people with anxiety and its expected increase in prevalence, even in populations living in areas distant to the conflict, such as Portugal.

## Data availability statement

The datasets presented in this article are not readily available because Ethics Committee restrictions. Requests to access the datasets should be directed to FP, filipeprazeresmd@gmail.com.

## Ethics statement

The studies involving human participants were reviewed and approved by Ethics Committee of the University of Beira Interior – Portugal (CE-UBI-Pj-2022-038-ID1367). The patients/participants provided their written informed consent to participate in this study.

## Author contributions

FP contributed to the study’s conception and design. FP, TM, IL, PS, and JS wrote the first draft of the manuscript and contributed to the interpretation of the results. TM was involved in data analysis. All authors contributed to the article and approved the submitted version.

## Funding

This article was supported by National Funds through FCT – Fundação para a Ciência e a Tecnologia, I.P., within CINTESIS, R&D Unit (reference UIDB/4255/2020) and within the scope of the project RISE, Associated Laboratory (reference LA/P/0053/2020).

## Conflict of interest

The authors declare that the research was conducted in the absence of any commercial or financial relationships that could be construed as a potential conflict of interest.

The reviewer VC declared a shared affiliation with the authors FP and JS to the handling editor at the time of review.

## Publisher’s note

All claims expressed in this article are solely those of the authors and do not necessarily represent those of their affiliated organizations, or those of the publisher, the editors and the reviewers. Any product that may be evaluated in this article, or claim that may be made by its manufacturer, is not guaranteed or endorsed by the publisher.
